# Dorsolateral prefrontal cortex plays causal role in probability weighting during risky choice

**DOI:** 10.1038/s41598-022-18529-6

**Published:** 2022-09-27

**Authors:** Ksenia Panidi, Alicia Nunez Vorobiova, Matteo Feurra, Vasily Klucharev

**Affiliations:** 1 Centre for Cognition and Decision Making, Institute for Cognitive Neuroscience, HSE University, ul. Myasnitskaya 20, 101000 Moscow, Russian Federation; 2 Amsterdam School of Economics, University of Amsterdam, Amsterdam, The Netherlands

**Keywords:** Neuroscience, Cognitive neuroscience, Decision, Human behaviour

## Abstract

In this study, we provide causal evidence that the dorsolateral prefrontal cortex (DLPFC) supports the computation of subjective value in choices under risk via its involvement in probability weighting. Following offline continuous theta-burst transcranial magnetic stimulation (cTBS) of the DLPFC subjects (N = 30, mean age 23.6, 56% females) completed a computerized task consisting of 96 binary lottery choice questions presented in random order. Using the hierarchical Bayesian modeling approach, we then estimated the structural parameters of risk preferences (the degree of risk aversion and the curvature of the probability weighting function) and analyzed the obtained posterior distributions to determine the effect of stimulation on model parameters. On a behavioral level, temporary downregulation of the left DLPFC excitability through cTBS decreased the likelihood of choosing an option with higher expected reward while the probability of choosing a riskier lottery did not significantly change. Modeling the stimulation effects on risk preference parameters showed anecdotal evidence as assessed by Bayes factors that probability weighting parameter increased after the left DLPFC TMS compared to sham.

## Introduction

Many of our everyday choices involve risk. One of the simplest examples of choice under risk is a decision to buy a lottery ticket. A person may choose to forego a fixed amount of money for a small chance to win a larger amount. Two key components of this choice that may affect the decision are the subjective value of money and the probability of winning (if it is known). Many recent studies suggest that both monetary rewards and outcome probabilities impact the decisions non-linearly. Combining non-invasive Brain Stimulation (NIBS) protocols such as transcranial magnetic stimulation (TMS) with structural modelling of risk preferences allows to elucidate the underlying neural mechanisms of the non-linearity of their impact and how their interaction results in a final decision to take or not to take a risk. In the present study, which employed TMS, we tested the hypothesis that the dorsolateral prefrontal cortex (DLPFC) is involved in decisions under risk due to its involvement both in assessing the value of money and in non-linearity of probability weighting.

Expected utility theory assumes that the marginal value of money decreases, which corresponds to the observation that the majority of people are risk averse, while probability weighting is linear^1^. Alternative theories of choice under risk that have found significant empirical support, such as Prospect theory and Rank-dependent utility theory, postulate that marginal utility of money decreases in the gain domain and increases in the loss domain, while probability weighting is non-linear^2,3^. One famous example demonstrating that probability has a non-linear impact on risky choice is the Allais paradox, where adding a 1 percentage point probability of obtaining zero has a different impact on participants’ choices depending on whether it is added to a sure option or to an option that already contains some positive chance of obtaining nothing^4^. Typically, experimental participants tend to overweight small probabilities and underweight large probabilities, although a reverse pattern is sometimes observed as well^5^. Importantly, non-linearity in probability weighting is distinct from probability perception. Probability weights in risky choice represent the impact that outcome probabilities have on the lottery utility^6^. This impact may result from a distorted perception of probabilities, or from other sources, such as distorted attentional processes which lead to probabilities being integrated into the final decision in a non-linear fashion, while probability perception per se is unchanged^7^.

Functional neuroimaging studies have implicated the anterior insula, ventral striatum, anterior cingulate cortex, parietal cortex and prefrontal cortex in decisions under risk and uncertainty^8,9^. The DLPFC has been shown to be involved in decision making, particularly in making choices under risk^9^. Several studies have demonstrated that DLPFC activity might be correlated with the decision value of an option in a decision-making task^10–12^. Higher activation of the DLPFC predicted safer choices in a risky choice task^13^. A meta-analysis of fMRI studies demonstrated activation of the right DLPFC in decisions under risk^9^. In addition, it reported that the DLPFC is more likely to be activated bilaterally in situations where a risky choice had to be made (decision risk) than in situations where a realization of a risky outcome was observed without making a choice (anticipation risk).

Several studies have indicated that DLPFC activity may be linked to individual components of risk taking such as reward value^14^ and probability^15^. Activity in a subregion of the DLPFC has also been correlated with reward magnitude, probability and expected value^16^. Additionally, DLPFC activity has been correlated with the subjective value of a lottery^17^, which suggests that the DLPFC may be involved in both reward value and probability.

Studies employing NIBS methods have demonstrated that down- or up- regulation of DLPFC excitability may lead to changes in risk preferences. For example, risk appetite was shown to increase after inhibitory TMS over the right (but not left) DLPFC^18^. A recent study showed that unilateral transcranial direct current stimulation (tDCS) over the DLPFC led to significant changes in risk preferences, asymmetrically between gains and losses^19^. These results are to some extent paralleled by findings that anodal tDCS stimulation of the DLPFC leads to more risk taking in the gain domain and to less risk taking in the loss domain^20^. Lastly, some studies have indicated that tDCS over the DLPFC results in safer choices^21,22^, while others have found the opposite effect^23,24^.

Although these studies collectively suggest that the DLPFC plays a major role in making risky choices, a question remains as to whether its activity mediates risky choice via probability weighting, via marginal utility (value) of monetary outcomes, or both.

In the present study we causally address the hypothesis that the DLPFC is involved both in the subjective valuation of a monetary reward and in probability weighting. The hypothesis was not preregistered but was formulated prior to the collection of the data. The hypothesis was well grounded in the existing literature on the role of DLPFC in risk taking. Several previous studies mentioned the possible role of the lateral PFC in separate components of choice under risk, such as reward magnitude, reward probability, and expected value^14,25,26^. However, previous studies including those exploring causal role of the DLPFC in risky choice with non-invasive brain stimulation were not focusing on the estimation of the risk preference parameters but rather on observing changes purely on a behavioural level. Therefore, in the present study we used an experimental design that is typically employed in economic studies estimating risk preference parameters^27^. We combined offline repetitive TMS over the left and right DLPFC and sham over the right DLPFC, performed in a randomized and counterbalanced order, with a random lottery pair (RLP) task, which is widely used in economics to estimate the degree of risk aversion as well as the curvature of the probability weighting function on an individual level.

Following offline TMS, subjects completed a computerized task consisting of 96 binary lottery choice questions presented in random order. Using the hierarchical Bayesian modeling approach, we then estimated the structural parameters of risk preferences (degree of risk aversion and the curvature of the probability weighting function) and analyzed the obtained posterior distributions to determine the effect of stimulation on model parameters.

We find that in the gain domain, downregulation of the left DLPFC excitability significantly decreases the likelihood of choosing an option with higher expected value controlling for the difference in standard deviations. At the same time, we do not find a significant difference in the probability of choosing an option with higher standard deviation. We further estimate the parameters of risk preference using a hierarchical Bayesian approach. We find that the left DLPFC TMS induced a significant increase in the probability weighting parameter on the group level which implies more distorted probability weights.

Overall, these data provide evidence that the DLPFC is involved in integrating different aspects of a decision related information to determine the value of each option. In particular, this is the first study to demonstrate the causal involvement of the DLPFC in probability weighting during risky choice.

## Materials and methods

### Participants

A total of 30 healthy volunteers (56% females, mean age = 23.6, min age = 18, max age = 34) participated in all three sessions of the experiment. Participants were recruited via paper flyers distributed on the university campus as well as advertisements on the Internet. The exclusion criteria described below were not preregistered but are typical for transcranial magnetic stimulation and decision-making studies. Potential subjects were queried about their area of education, and those with prior knowledge of economics or technical sciences (math, physics, computer science, etc.) were not invited to participate. These subjects were excluded due to possible knowledge of various theories of choice (e.g., Expected Utility, Prospect Theory, etc.) that might bias the outcome—they may try to deliberately align their behavior with these theories or may engage in calculating mathematical expectation of lotteries. Unlike students majoring in Economics or technical sciences, Psychology students were allowed to participate. Importantly, Behavioral economics is not a part of the local Psychology students’ curriculum.

Other exclusion criteria included regular sleep of less than 6 h per day, self-reported left-handedness, past history of brain injury or head trauma, being diagnosed with any psychiatric or neurological illness including epilepsy and migraines, family history of epilepsy, taking any prescribed medication, and having metal objects inside the body. All participants read and signed the informed consent form prior to the experiment. All procedures were approved by the ethics committee of the National Research University-Higher School of Economics (HSE), Moscow. All experimental procedures were performed in accordance with relevant guidelines and regulations.

Out of the 30 participants the behavioral data of two subjects were not included in the analysis due to a large interhemispheric difference in the motor threshold (more than 10 percentage points of the maximum stimulator output). Since we used sham stimulation only on the right DLPFC it is crucial to perform sham and real stimulation with similar intensity. Otherwise, placebo effects on one of the hemispheres or an insufficient stimulation on the other may bias participants’ behavior. For one of the excluded participants the left motor threshold was substantially lower than the right (left resting motor threshold (RMT) = 57%, right RMT = 67%), which could have led to insufficient stimulation intensity on the left DLPFC. The other participant in the post-experimental debriefing reported being right-handed but learned to write with the left hand following the right hand injury. This might have led to insufficient stimulation intensity on the right DLPFC (right RMT = 28%, left = 38%). The average difference in motor thresholds for the remaining participants was − 0.15 percentage points (p value = 0.82). As a robustness check we performed the same data analysis using the whole dataset, which showed that results did not qualitatively differ from those obtained on the restricted sample (see Supplemental Materials).

### Experimental task and payment

**Figure 1:**
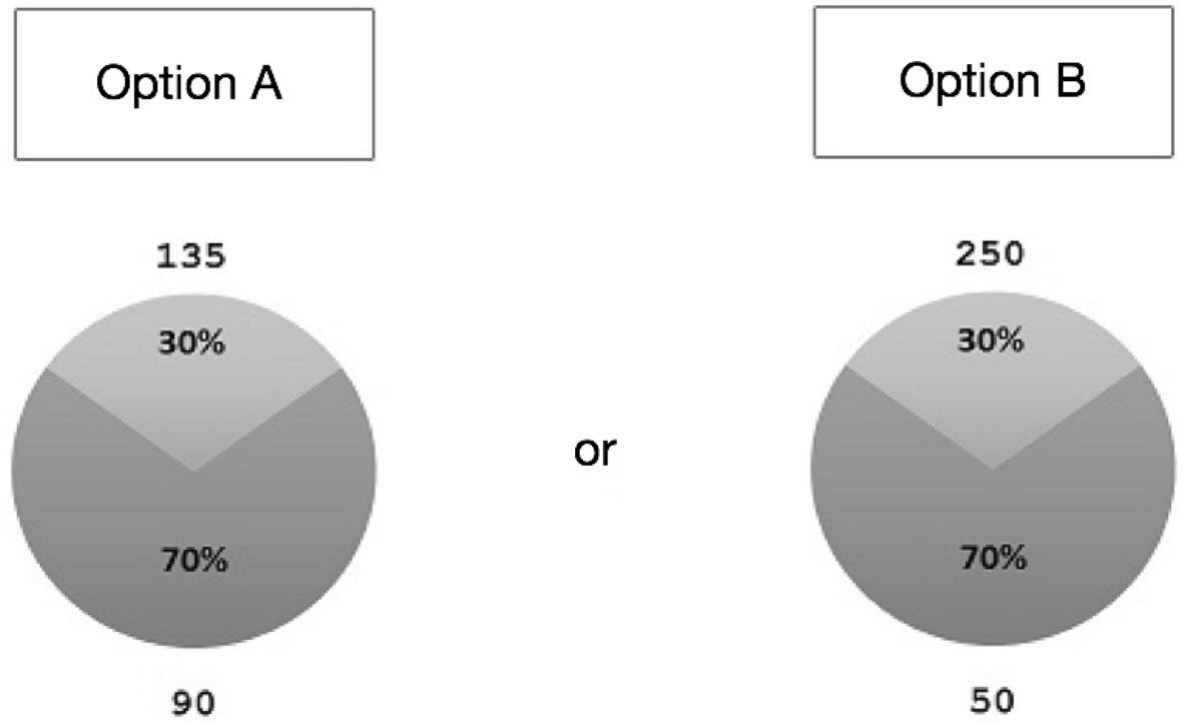
Task design. Subjects had to indicate their preferred option by pressing one of two buttons on the keyboard. The diagrams graphically and numerically present probability distributions for each lottery as well as the corresponding lottery outcomes (in monetary units).

The experimental task consisted of 96 self-paced binary lottery choice questions. Each question involved a choice between option A and option B, where each option represented a lottery. Figure 1 presents an example of a screen subjects would see during the experiment. A participant had to indicate which lottery they would prefer to play by pressing one of two buttons on the keyboard located in front of them (indifference between lotteries was not allowed).

After making their choice, a blank screen with a fixation cross in the middle would appear, which would automatically switch to the next question after 500 ms.

In all questions, outcome probabilities were the same between the two lottery options. Half of the questions were formulated purely in the gain domain and the other half purely in the loss domain (no mixed lotteries were offered). In each domain, the experimental task consisted of the three multiple price lists (MPL) similar to those used in other studies to detect changes in risk preferences^28^. Each MPL represented a list of 16 binary choice questions ordered by probability of the best outcome ranging from 0 to 1 (presented to the participants in random order). An example of an MPL used in the task is seen in Table 1. The monetary amounts were chosen to make the lottery outcomes meaningful enough for the participants who were predominantly university students. Another consideration was to be able to capture a range of risk preferences typically observed in these experiments with the risk aversion coefficient for the CRRA function between -1 and 1. Finally, the amounts used were in the range similar to that in^28^ taking into account the conversion rate between monetary units and USD at the time of data collection.

**Table 1:** Example of the MPL used in the experimental task.

Number of question	Option A		Option B		Probability of best outcome	Probability of worst outcome	Expected value of Option A	Expected value of Option B
	Outcome 1	Outcome 2	Outcome 3	Outcome 4				
1	260	180	350	50	0	1	180	50
2	260	180	350	50	0.01	0.99	180.8	53
3	260	180	350	50	0.05	0.95	184	65
4	260	180	350	50	0.10	0.90	188	80
5	260	180	350	50	0.15	0.85	192	95
6	260	180	350	50	0.20	0.80	196	110
7	260	180	350	50	0.30	0.70	204	140
8	260	180	350	50	0.40	0.60	212	170
9	260	180	350	50	0.50	0.50	220	200
10	260	180	350	50	0.60	0.40	228	230
11	260	180	350	50	0.70	0.30	236	260
12	260	180	350	50	0.80	0.20	244	290
13	260	180	350	50	0.85	0.15	248	305
14	260	180	350	50	0.90	0.10	252	320
15	260	180	350	50	0.95	0.05	256	335
16	260	180	350	50	1	0	260	350

Expected values were not shown on the screen.

For the first several questions of each MPL, lottery A had a higher expected value (EV) than lottery B, while in all other questions the opposite was true. If a participant is risk-neutral, she will choose option A in all cases where it delivers higher EV and option B otherwise. However, if a participant demonstrates risk aversion, she will switch to option B later in the list.

Here, option A has a greater expected value than option B for the first 9 rows. If a participant is risk neutral she would choose option A in questions 1 through 9 and option B in questions 10 through 16. If a participant is risk-averse, she will also prefer option A for lotteries from 1 to 9 as well as for some lotteries from 10 to 16, depending on the degree of risk aversion. Therefore, her choice will coincide with risk-neutrality for some of the questions in the list but not for all of them.

All 96 lottery pairs were presented in a randomized order (random lottery pair design). The complete list of lotteries can be found in Supplemental Materials. In each pair of lotteries, the probabilities of the corresponding high and low outcomes were identical. The ordering of lotteries on the screen was also randomized: in half of the questions the lottery with a higher variance appeared as option A, while in the other half it appeared as option B. Finally, the lottery questions in the loss domain were symmetrical to those in the gain domain (i.e., only the sign of outcomes changed from positive to negative). To minimize the possibility that subjects would remember their answers from previous sessions, the order of the questions was randomized and unique in each session. The positions of the monitor and keyboard were adjusted to suit each participant prior to the beginning of the task. To eliminate possible effects of time pressure on risk preferences found in previous studies^29^, subjects were told that they had unlimited time for the task.

Participants were paid a 500 monetary unit (MU) participation fee (~ 8.7 USD based on an official exchange rate) for each session and were informed that all payments would be administered at the very end of the third session. Monetary units corresponded to the local currency and this was explicitly stated in the instructions, so that participants were well informed about the amount of money they will receive. Additionally, they were informed that one question from each session would be selected randomly and a lottery that was chosen for this particular question would be played out for real to determine the final payment the participant would receive for each session. Participants could win or lose money depending on the selected question. Therefore, the earned amount would be added or subtracted from the 500 monetary units received for the corresponding session. Conversion using the BigMac index at the time of the data collection indicates that the purchasing power of 500 MU was equivalent to 21.19 USD.

The participants were also informed that they would not learn the monetary outcome of each session until the end of the third session. This restriction was introduced to avoid the influence of any past outcomes on subjects’ risk preferences in further sessions^30,31^. On average, participants earned 25.5 MU in total for three sessions on top of the participation fee. The average amount was relatively small since the pure gain and pure loss lotteries were symmetrical and only one question was selected for payment for each session. In all sessions, completion of the task took 10.5 min on average. In a typical risky choice task the response time varies between 3 and 10 s. For example, in^13^ participants had unlimited time to indicate their choice and the average response time was only 2.5 s. In^32^ the choice options were presented on the screen for 5 s. In^33^ the average reaction time was 3.84 s. Therefore, our participants had spent sufficient time to make a conscious decision. Additionally, in the debriefing stage none of the participants indicated that the task was meaningless to them, that they did not understand the task or that they made thoughtless choices.

### Experimental design and stimulation protocol

For each participant, the experiment consisted of three sessions carried out on different dates separated by 3–4 days. Each session included one of the three treatments: (1) continuous theta-burst stimulation (cTBS) of the right DLPFC (“right”), (2) cTBS of the left DLPFC (“left”), (3) sham stimulation of the right DLPFC (“sham right”). The order of these treatments was randomized and counterbalanced between participants. To improve precision when positioning the coil, we employed a neuronavigation system which utilized the structural T1-weighted MRI scans that subjects obtained on a separate day prior to the experiment.

We used a perturbation neuronavigated cTBS protocol. cTBS is an advanced patterned TMS protocol which has been shown to be effective in modulating the cortical excitability of a specific brain area both in motor and cognitive domains^34^. Specifically, cTBS has been shown to induce suppression of cortical excitability^34^ and has been successfully used in studies of decision-making to explore reinforcement learning^35^, social preferences^36^, impulsivity^37^, and gambling behavior^38^.

The stimulation was performed using a figure-of-eight (75 mm diameter) Cool-B-65 coil through a MagVenture stimulator (MAGPRO R30 with MagOption, MagVenture, Inc.). The off-line stimulation paradigm was used; that is, stimulation was administered prior to performing the task. Stimulation intensity was set at 80% of the resting motor threshold (RMT) determined for each individual at the beginning of each session. The RMT was determined as the stimulation intensity inducing at least five motor evoked potentials (MEPs) of at least 50 µV out of 10 pulses on the motor hotspot of the *first dorsal interosseous* muscle^39^ in the hand contralateral to the side of DLPFC stimulation. The cTBS stimulation lasted 40 s. The coil was held tangentially to the scalp at a 45-degree angle to the midsagittal axis of the subject’s head. Subjects were given a 5-min break after the stimulation and before performing the task to allow for the downregulating effects of cTBS to take place^34^. Previous research has shown that this stimulation protocol downregulates the cortex for up to 60 min following stimulation^34^.

Sham (i.e., placebo) sessions were conducted in exactly the same way as regular sessions except for the way in which the coil was placed on the head. It was administered by placing the coil upside down on the participant’s head so that the magnetic field was directed away from the subject’s skull. The thickness of the coil allowed for a distance between the stimulation surface of the coil and the skull. This method of sham stimulation is frequently used in TMS research^40–42^ as it allows to maintain the whole stimulation procedure identical to the sessions with active stimulation including similar sound effects for the participants.

Stimulation protocols were run with online neuronavigation (Localite GmbH, Germany).

**Figure 2:**
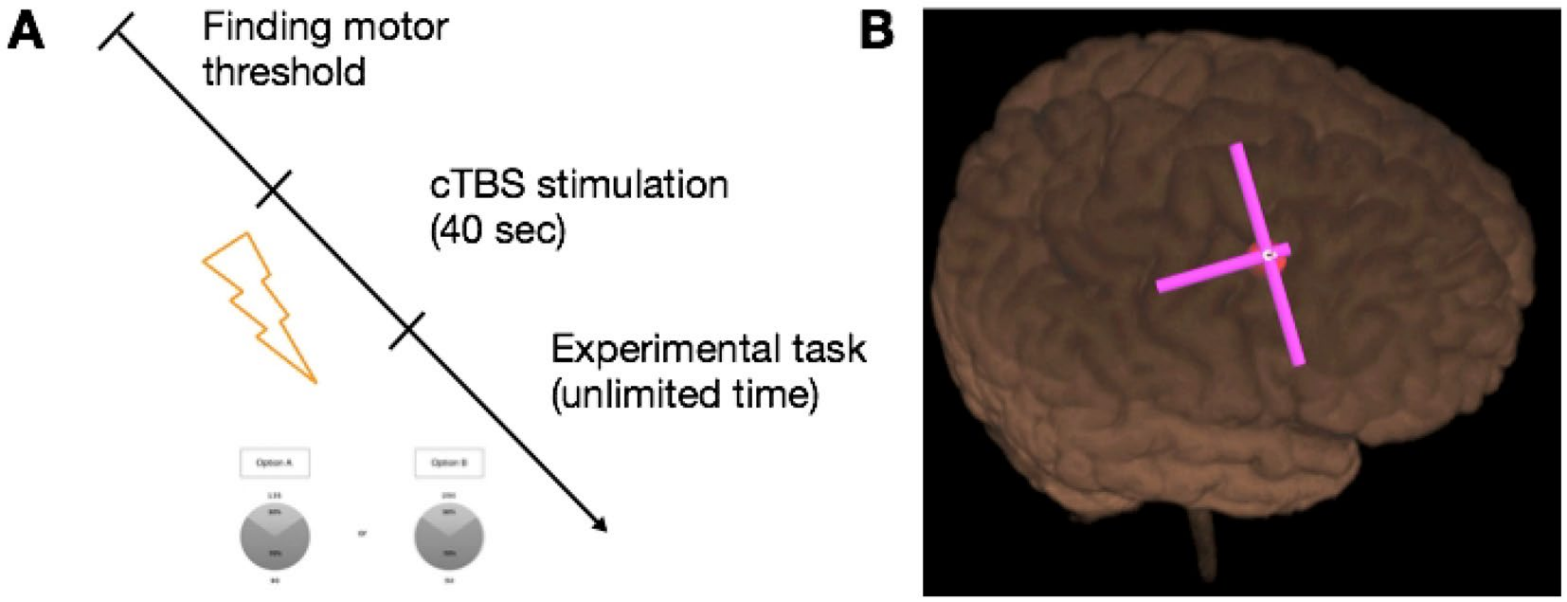
Experimental design. (**A**) experimental timeline. (**B**) example of coil positioning on the head (screenshot of the Localite neuronavigating system software).

Stimulation site coordinates were identified for each subject based on their T1-weighted structural MRI images. Montreal Neurological Institute (MNI) stereotaxic coordinates were back-normalized to subjects’ native brain space using an SPM8 toolbox (http://www.fil.ion.ucl.ac.uk/spm/software/spm8/). The MNI coordinates were selected based on the previous fMRI study, which revealed the peak activity of the DLPFC (right DLPFC (8, 18,44); left DLPFC (− 42, 16, 42)) correlated with subjective value of a lottery^17^. In the same study, activity in a similar region of the DLPFC correlated with expected return and subjective expected return of the lottery. Additionally, activity at similar coordinates of the right DLPFC was found to be correlated with decision risk as opposed to anticipation risk^9^ (right DLPFC (38, 22, 36)). TMS stimulation sites were identified on each participant’s scalp using the MRI-based Localite TMS Navigator system (Localite GmbH, Germany). Figure 2 presents the experimental timeline and an example of coil positioning during the stimulation.

### Behavioral analysis

A traditional way to analyze behavioral data from the MPL experiments implies the analysis of a shift in a crossover point in the MPL list. However, when the ‘random lottery pair’ design is introduced many people make inconsistent choices, having several switching points within one MPL list. Therefore, to analyze the behavioral effects of TMS we focus on the trial-by-trial probability of choosing a riskier lottery (i.e., a lottery with higher standard deviation), and the probability of choosing a lottery with higher expected reward. In both cases we estimate the linear mixed model including the dummy variables for the TMS stimulation condition as the main variables of interest, as well as a number of control variables, with a logit link function and subject-level random effects. Both types of regressions included the difference in standard deviations between lotteries, difference in means, self-reported level of discomfort, and the trial number to control for fatigue effects.

Next, to take into account the differences in outcomes and probabilities across trials in a non-linear way as well as to directly incorporate the inconsistency of subjects’ answers we used a structural modelling approach, described below^43^.

### Structural modelling of risk preferences

To determine the effect of TMS of the DLPFC on risk preference parameters, we estimate a stochastic model of choice assuming the rank-dependent utility function with probability weighting and the logistic distribution of the random error (*Luce* form)^43^. The model assumes the utility function with constant relative risk aversion (CRRA), which is commonly used in economics:

1 $$U\left(x\right)=\left\{\begin{array}{c}{x}^{r}, \quad x\ge 0\\ -{\left(-x\right)}^{r}, \quad x<0\end{array}\right.$$

where $x$ represents a monetary outcome of a lottery and $r$ represents the coefficient of risk preferences. In the gain domain, $r<1$ corresponds to risk aversion and $r>1$ indicates risk seeking, while the interpretation is reversed in the loss domain. Therefore, greater values of $r$ indicate higher (lower) risk tolerance in gains (losses).

For a two-outcome lottery, the rank-dependent utility model assumes that subjects weight the probability of the best outcome in a non-linear way^44^, while the rest of the weight is given to the worst outcome as probability weights sum up to 1. The subject’s choice is then driven by the weighted subjective utility of option $k\in \left\{A, B\right\}$ determined in the following way:

2 $$E{U}_{k}={w(p}_{1}^{k})U\left({x}_{1}^{k}\right)+\left(1-w\left({p}_{2}^{k}\right)\right)U\left({x}_{2}^{k}\right)$$

where ${x}_{1}^{k}>{x}_{2}^{k}$ and ${w(p}_{1}^{k})$ is a probability weight given to the best outcome. We tested three specifications of the probability weighting function—linear, 1-parameter Prelec function and 1-parameter Kahneman and Tversky^3^ function. Table 2 summarizes these model specifications.

**Table 2:** Stochastic choice model specifications used for parameter estimation.

Model index	Utility assumptions	Probability weighting function	Model	Source
(1)	Linear	$w\left(p\right)=p$	Linear	
(2)	Rank-dependent	$w\left(p\right)=\frac{{p}^{\gamma }}{{\left({p}^{\gamma }+{\left(1-p\right)}^{\gamma }\right)}^{\frac{1}{\gamma }}}$	Kahneman–Tversky	Tversky and Kahneman (1992)
(3)	Rank-dependent	$w\left(p\right)={\mathrm{e}}^{-{(-\mathrm{ln}p)}^{\alpha }}$	Prelec–1	Prelec (1998)

Parameters $\gamma$ and $\alpha$ correspond to the curvature (degree of distortion) of the probability weighting function.

Additionally, we assume a strict utility model which implies that subjects choose between lotteries on the basis of the difference in the logarithms of expected utilities^45^. To reduce the correlation between model parameters $r$ and $\mu$ we apply the value function transformation as suggested in previous studies^46–48^. This specification translates into the probability of a subject choosing option A over option B equal to:

3 $$P\left(A\succ B \right)=\frac{1}{1+\mathrm{exp}\left(-\frac{1}{\mu }\cdot \frac{1}{r}\cdot \mathit{ln}\left(\frac{E{U}_{A}}{E{U}_{B}}\right)\right)}$$

where $\mu$ is a ‘noise’ parameter, and $EU$ stands for the expected utility of an option. Variable $\tau =\frac{1}{\mu }$ then signifies inverse temperature and indicates consistency of choices. When $\mu$ tends towards zero (and $\tau \to \infty$), the model becomes deterministic; that is, the agent chooses the lottery that provides a greater expected utility. Therefore, greater values of $\tau$ correspond to more consistent choices, while lower values indicate more randomness.

To analyze the effect of TMS on risk preferences, we employ a hierarchical Bayesian modelling approach which constitutes a compromise between complete pooling of individual data and complete separation^49^.

Each risk preference parameter was modelled as a combination of its baseline level and a change produced by TMS in the following way:

4 $$\begin{aligned} r_{i} = & r_{i}^{0} + \Delta r_{i}^{{right}} \cdot I\left( {TMS_{{right}} } \right) + \Delta r_{i}^{{left}} \cdot I\left( {TMS_{{left}} } \right) \\ \gamma _{i} = & \gamma _{i}^{0} + \Delta \gamma _{i}^{{right}} \cdot I\left( {TMS_{{right}} } \right) + \Delta \gamma _{i}^{{left}} \cdot I\left( {TMS_{{left}} } \right) \\ \tau _{i} = & \tau _{i}^{0} + \Delta \tau _{i}^{{right}} \cdot I\left( {TMS_{{right}} } \right) + \Delta \tau _{i}^{{left}} \cdot I\left( {TMS_{{left}} } \right) \\ \end{aligned}$$

where $I(\cdot )$ equals 1 for trials from the corresponding TMS condition and 0 otherwise. For a linear probability weighting model, the equation for $\gamma$ was omitted. We use the following weakly informative priors for the group-level parameters:

5 $$\begin{aligned} & \mu _{{r^{0} }} \sim N\left( {0,5} \right) \\ & \mu _{{\gamma ^{0} }} \sim N\left( {1,3} \right) \\ & \mu _{{\tau ^{0} }} \sim N\left( {0,10} \right) \\ & \mu _{{\Delta r^{{right}} }} ,\mu _{{\Delta r^{{left}} }} ,\mu _{{\Delta \gamma ^{{right}} }} ,\mu _{{\Delta \gamma ^{{left}} }} ,\mu _{{\Delta \tau ^{{right}} }} ,\mu _{{\Delta \tau ^{{left}} }} \sim N(0,1) \\ \end{aligned}$$

The standard deviations of all group parameters were sampled from a lognormal distribution with mean 0 and standard deviation of 3. The hierarchical model structure was set assuming each individual parameter being normally distributed with the mean and standard deviation equal to the group-level mean and standard deviation of that parameter. All parameters were sampled from an unconstrained space and then transformed to a constrained space using the exponential transformation for positively defined parameters and a Phi-transformation for parameters with two-sided boundaries. We imposed the following restrictions on the individual and group parameter space: ${r}_{i}^{0}\in (0, 5)$,${\gamma }_{i}^{0}\in (0, 6)$, ${\tau }_{i}^{0}\in (0,+\infty )$, $\Delta {r}_{i}^{right,left}\in (-\mathrm{2,2})$, $\Delta {\gamma }_{i}^{right, left}\in (-\mathrm{1.5,1.5})$, $\Delta {\tau }_{i}^{right, left}\in (-\mathrm{5,5})$. The intervals for parameters indicating TMS effects were chosen to be wide enough to allow exploration of rather big TMS effects relative to the baseline parameter levels typically observed in previous risk-preference studies. However, since baseline parameter values are on slightly different scales, the allowed intervals also differed. For example, as baseline consistency parameter ${\tau }_{i}^{0}$ can be much larger than risk aversion parameter ${r}_{i}^{0}$ (which rarely is much higher than 3 in an experimental setting), a larger interval was allowed for $\Delta {\tau }_{i}^{right, left}$ than for $\Delta {r}_{i}^{right,left}$. The obtained posterior distributions showed that all posterior samples belonged well within these intervals and did not approach the boundaries.

The sampling was performed using the Markov Chain Monte Carlo (MCMC) method (NUTS algorithm) with 8 chains each containing 1000 iterations for a warm-up and additional 4000 iterations for sampling from posterior distribution giving 32,000 posterior samples for each parameter. Convergence for all three models was confirmed using the visual inspection of the traceplots and the $\widehat{R}$ statistics. The max $\widehat{R}$ value for the group-level parameters equaled 1.0009 for the linear model, 1.001 for the Prelec-1 model, and 1.006 for the Kahneman-Tversky model, indicating that chains have mixed well. To select the best-performing model we calculated the LOOIC and WAIC criteria. According to both of these criteria, Kahneman-Tversky model outperformed the other models in terms of the goodness of fit (see Table 3). Effective sample size for the selected model group-level parameters was 8812 for ${\mu }_{{r}^{0}}$, 7754 for ${\mu }_{{\gamma }^{0}}$, 14,053 for ${\mu }_{{\tau }^{0}}$, 4048 for ${\mu }_{{\Delta r}^{right}}$ , 8358 for ${\mu }_{{\Delta \gamma }^{right}}$, 16,234 for ${\mu }_{{\Delta \tau }^{right}}$, 8723 for ${\mu }_{{\Delta r}^{left}}$, 8631 for ${\mu }_{{\Delta \gamma }^{left}}$, and 14,825 for ${\mu }_{{\Delta \tau }^{left}}$.

**Table 3:** LOOIC and WAIC information criteria to determine the best fitting model.

Model	LOOIC	WAIC
Linear	2516.7	2509.15
Non-linear (Prelec-1)	2632.1	2703.20
Non-linear (KT)	2250.0	2236.72

Posterior predictive check was performed for the selected model by obtaining 8000 random parameter samples from the joint posterior distribution. The proportion of correctly fitted choices was calculated for each selected sample to obtain the probability that the model fits participants’ choices correctly. This analysis indicated that the selected model fitted the participants’ choices correctly credibly better than chance (median 0.846, 95% CI [0.836, 0.857]). Additionally, we performed parameter recovery procedure for this model which indicated that the group-level parameters of interest can be recovered well (see Supplemental Materials). As there is an ongoing discussion regarding the appropriate decision thresholds in Bayesian hypothesis testing^50,51^, in the analysis we provide the posterior distributions of the parameters of interest as well as both 95% and 89% credible intervals.

Next, we analyzed the posterior distributions of the group-level parameters to determine the effects of TMS on risk preferences. All computational procedures were carried out with R software (version 3.5.0, the R Project for Statistical Computing, Vienna, Austria) and the *rstan* package (version 2.19.3) for R.

## Results

### Behavioral analysis

We first present the results of a behavioral analysis for the probabilities of choosing a lottery with higher standard deviation and with higher expected reward. Previous research suggested that decision-making mechanisms for gains and losses may differ^52–54^, which may lead to people exhibiting different risk preferences depending on the valence of the outcomes, both in terms of the value function curvature and probability weighting^55,56^. Thus, we perform behavioral analysis and report the results separately for gains and losses. Table 4 presents the estimation results. Control variables included the difference in standard deviations in favor of a more risky lottery, the difference in means in favor of a more risky lottery, the trial number to control for tiredness of participants, and the self-reported level of discomfort during the stimulation, as well as the interaction terms of these variables with the TMS stimulation dummies. None of the interaction terms, except the interaction with discomfort level, were significant and were, hence, excluded from the set of explanatory variables.

**Table 4:** Effects of the right/left DLPFC TMS on the behaviour in a binary lottery choice task on a trial-by-trial level relative to sham.

Dependent variable	Probability of choosing a higher SD lottery		Probability of choosing a higher EV lottery	
	Gains	Losses	Gains	Losses
TMS (right DLPFC)	− 0.123 (0.245)	0.002 (0.219)	− 0.001 (0.242)	0.039 (0.247)
TMS (left DLPFC)	− 0.056 (0.243)	0.004 (0.218)	− 0.518* (0.227)	− 0.343 (0.229)
Discomfort	− 0.156 (0.095)	0.140 (0.085)	− 0.179* (0.087)	− 0.129 (0.085)
Trial	− 0.002 (0.002)	0.001 (0.002)	− 0.001 (0.002)	− 0.001 (0.002)
Difference in std.dev	0.022*** (0.002)	0.006*** (0.001)	− 0.038*** (0.002)	− 0.027*** (0.002)
Difference in means	0.015*** (0.002)	0.010*** (0.001)	− 0.001 (0.002)	0.0003 (0.002)
TMS (right DLPFC) × discomfort	0.145 (0.107)	− 0.063 (0.096)	0.104 (0.104)	0.022 (0.105)
TMS (left DLPFC) × discomfort	0.111 (0.099)	− 0.096 (0.090)	0.283** (0.095)	0.130 (0.092)
Difference in std.dev. × difference in means	0.0005*** (0.00003)	0.0004*** (0.00002)	− 0.0002*** (0.00003)	− 0.0001** (0.00003)
Observations	4032	4032	4032	4032
Log likelihood	− 1286.095	− 1576.840	− 1410.605	− 1366.499
Akaike Inf. Crit	2594.190	3175.680	2843.211	2754.997

All regressions are mixed-effect generalized linear models with a logit link function and subject-level random effects. Columns 1 and 2 use probability of choosing a riskier (higher SD) lottery as a dependent variable. Columns 3 and 4 use probability of choosing a lottery with higher expected reward (higher EV) as a dependent variable. ***p < 0.001; **p < 0.01; *p < 0.05. Standard errors in parentheses.

Among various regression specifications tested, the best-performing models according to the AIC and BIC criteria showed that TMS had no significant effect on the probability of choosing a riskier lottery, however the left DLPFC TMS led to significantly lower probability of choosing an option with higher expected reward (p value = 0.027).

Regression analysis in the loss domain did not reveal any effect of TMS stimulation on the propensity to choose a riskier option or an option with higher expected reward.

We further investigate the effects of TMS at the level of risk-preference parameters. Since we did not find any behavioral effects of stimulation on losses the hierarchical Bayesian modelling presented below focuses on the analysis in the gain domain. The structural modelling results demonstrating lack of effects in the loss domain may be found in the Supplemental Materials.

### Estimation of risk-preference parameters

**Figure 3:**
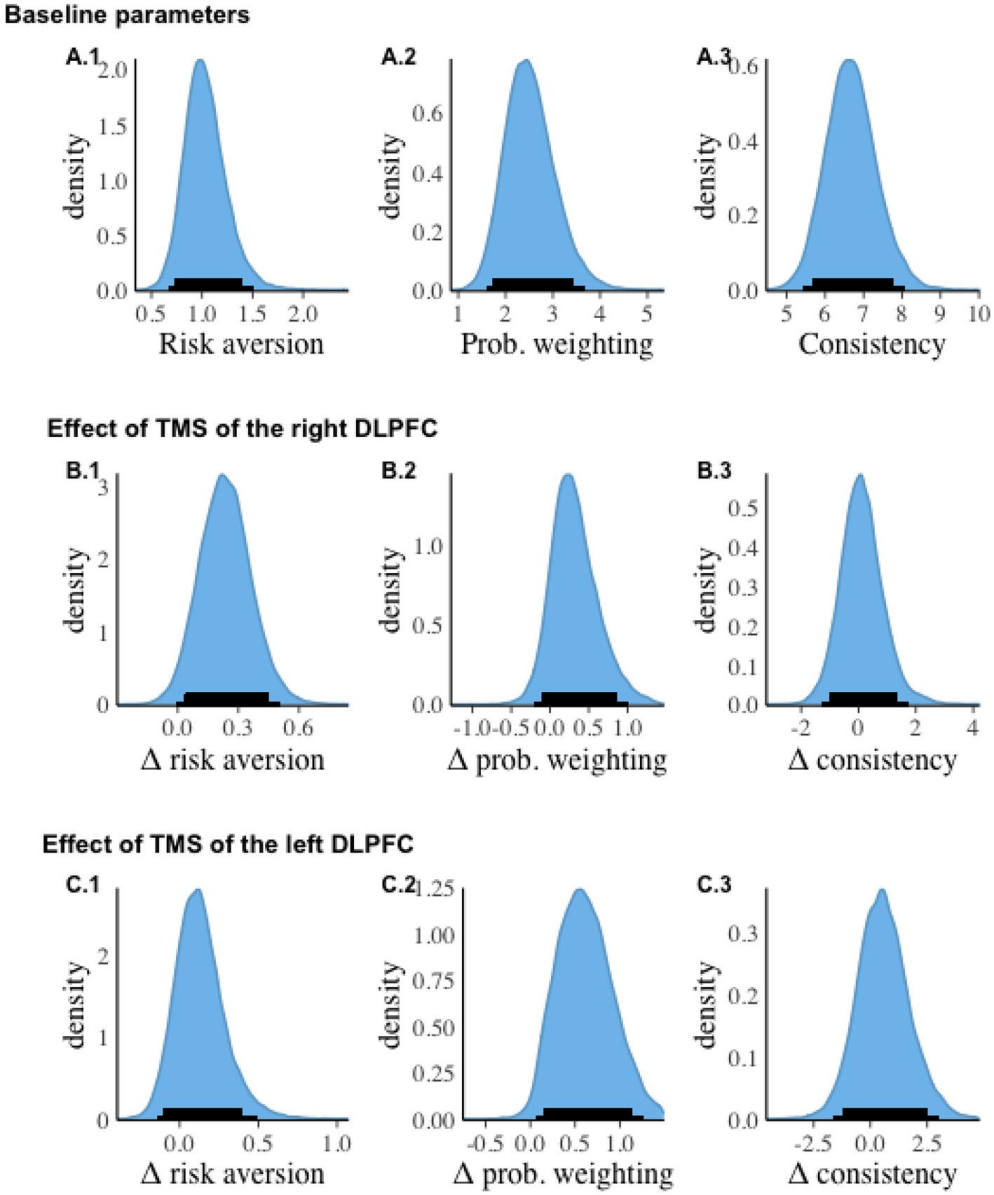
Baseline risk preference parameters and their changes after the DLPFC TMS in the *gain domain*. Baseline risk preference parameters: (**A.1**) risk aversion (${\mu }_{{r}^{0}}$), (**A.2**) probability weighting (${\mu }_{{\gamma }^{0}}$), **A.3** consistency (${\mu }_{{\tau }^{0}}$). Shift in risk preference parameters after right DLPFC TMS: (**B.1**) change in risk aversion (${\mu }_{\Delta {r}^{right}}$); (**B.2**) change in probability weighting (${\mu }_{\Delta {\gamma }^{right}}$); (**B.3**) change in consistency (${\mu }_{\Delta {\tau }^{right}}$). Shift in risk preference parameters after left DLPFC TMS: (**C.1**) change in risk aversion (${\mu }_{\Delta {r}^{left}}$); (**C.2**) change in probability weighting (${\mu }_{\Delta {\gamma }^{left}}$); (**C.3**) change in consistency (${\mu }_{\Delta {\tau }^{left}}$). The thin and thick black lines on the horizontal axis indicate the 95% and 89% CIs respectively.

In the gain domain, participants were almost risk-neutral at the group level with mean baseline risk aversion equal to ${\mu }_{{r}^{0}}$= 1.03, 95% CI [0.68, 1.49]. At the group level participants demonstrated significant distortion of probability in the baseline with the mean probability weighting parameter estimated at ${\mu }_{{\gamma }^{0}}$= 2.5, 95% CI [1.61, 3.67], and were consistent in their answers with ${\mu }_{{\tau }^{0}}$= 6.68, 95% CI [5.46, 8.06]. Figure 3 shows the sampled posterior distributions for the three baseline parameters, and the estimated change in parameters due to the stimulation, as well as their 95% CIs. Here and below all figures show the posterior distributions of the group-level parameters based on the 32,000 posterior draws. Multiple comparison correction was not applied since the hierarchical Bayesian modelling mitigates this problem^57^.

To characterize the effect of TMS on risk preference parameters, we provide the means, as well as 89% and 95% CIs for each parameter. Additionally, for each parameter of interest we use the Savage-Dickey ratio at zero to compute the Bayes factor for testing the hypothesis that the group-level parameter is different from zero (i.e., testing ${H}_{1}: \delta \ne 0$ against ${H}_{0}: \delta =0$). The results are summarized in Table 5.

**Table 5:** Summary the DLPFC TMS effects on risk preference parameters in the *gain domain*: mean, 89% and 95% CIs, and the Bayes Factor.

Parameter	Mean	89% CI	95% CI	BF
**Effects of the right DLPFC TMS**				
Δ risk aversion (${\mu }_{\Delta {r}^{right}})$	0.236	[0.039; 0.44]	[− 0.004; 0.50]	0.46
Δ prob. weighting (${\mu }_{\Delta {\gamma }^{right}})$	0.317	[− 0.11; 0.84]	[− 0.20; 1.00]	0.38
Δ consistency (${\mu }_{\Delta {\tau }^{right}}$)	0.114	[− 1.01; 1.34]	[− 1.27; 1.73]	0.17
**Effects of the left DLPFC TMS**				
Δ risk aversion (${\mu }_{\Delta {r}^{left}}$)	0.129	[− 0.095; 0.40]	[− 0.14; 0.49]	0.11
Δ prob. weighting (${\mu }_{\Delta {\gamma }^{left}}$)	0.61	[0.15; 1.14]	[0.081; 1.25]	2.81
Δ consistency (${\mu }_{\Delta {\tau }^{left}}$)	0.563	[− 1.17; 2.46]	[− 1.52; 2.96]	0.30

The Bayes factor indicates evidence in favor of ${H}_{1}:\delta \ne 0$ against ${H}_{0}:\delta =0$.

The results suggest that the left DLPFC TMS caused a credible increase in the probability weighting parameter (the 95% CI does not include zero). In our sample, the estimated baseline probability weighting parameter is greater than 1 ${(\mu }_{{\gamma }^{0}}$ = 2.5). In this range, a further increase in probability weighting parameter implies greater deviation from linearity, and therefore, more distortion.

No credible changes were observed in risk aversion coefficient or consistency of preferences. A positive shift in the risk aversion parameter after the right DLPFC stimulation is observed, however it is detected only at the 89% credibility level which indicates that the effect may be weak. The Bayes factor provides further evidence that the TMS stimulation of the left DLPFC shifted probability weighting parameter relative to baseline, although as BF is less than 3, the evidence is anecdotal. This parallels the conclusions derived from the 95% CI analysis where zero is only slightly away from the lower boundary of the credible interval.

## Discussion

Previous neuroimaging studies have shown that the DLPFC is involved in determining goal values, or assigning values to each option at hand, at the moment of making a choice^9,58,59^. However, an option may have higher or lower subjective value depending on the size of a potential reward as well as the probability of obtaining it^60,61^. Therefore, the DLPFC may potentially contribute to option valuations in a risky context via both of these components.

In the present study, we used neuronavigated TMS of the DLPFC to demonstrate its causal role in risky choice via its involvement in probability weighting, the evidence for which is found in the left DLPFC.

In our experiment, in the gain domain, participants’ choices after the left DLPFC stimulation were consistent with higher non-linearity of probability weighting, while we found no significant effect on the marginal value of money.

Previous studies that used NIBS methods to up or downregulate the excitability of the prefrontal cortex typically coupled brain stimulation with experimental tasks which did not allow for a separate determination of effects of stimulation on reward value and reward probability. One task that is frequently used to explore risk taking is the Balloon Analogue Risk Task (BART). In this task, subjects are repeatedly faced with the choice to either inflate a balloon, which simultaneously increases the amount of a cash prize and the likelihood of the balloon bursting, or to cash out with a smaller but certain outcome. However, the probability of explosion is usually unknown to the participants^62,63^. Therefore, the changes in behavior observed in this task as a result of non-invasive brain stimulation may potentially be due to changes in perceived reward value as well as participant’s *belief* regarding explosion probability, and not necessarily due to changes in probability distortions^64^. Another task that is often used in neuroeconomic studies on risk taking is the MPL task^28^. As was mentioned earlier, in this task changes in the crossover point, or the point after which participants switch their preference from safer to riskier lotteries, are analyzed to detect whether subjects behaved in a more or less risk averse manner following the brain stimulation^19,23^. However, on top of being a noisy measure of risk preferences, a shift in a crossover point alone would not indicate whether the observed behavioral changes resulted from differences in reward value or probability weighting.

The main advantage of our study is that by coupling the MPL with a random lottery pair design and implementing the structural modeling approach, we detect the TMS effects both on a behavioral level as well as on the level of risk preference parameters. However, the documented results indicate relatively weak effects and provide merely anecdotal evidence.

The interpretation of the present findings will depend on the framework through which the process of making a risky choice is viewed. Below we discuss it in light of two possible options: the framework suggested by the descriptive models of choice (such as expected utility, or Cumulative Prospect Theory (CPT) type of models), and the framework of heuristic rules in risky choice.

One common line of analysis (and the one used in our study) relies on descriptive models of choice such as expected utility, or cumulative prospect theory (CPT) where the value of each option is a combination of monetary outcomes weighted by the corresponding probabilities. If these descriptive models indeed accurately represent the valuation process during risky choice, then the obtained results suggest that the DLPFC disruption leads to the changes in this process or in its subcomponents. These may be the valuation of a monetary reward, the distorted perception of probabilities, and/or the process of integrating these components to assess the value of each option.

The obtained results are in line with previously mentioned findings that the DLPFC is involved in goal valuation in a situation of choice. Our results suggest that DLPFC might be involved in a more objective weighting of probabilities in a risky choice. Specifically, downregulation of the left DLPFC leads to a more non-linear probability weighting. These findings support the notion that the DLPFC plays a role in the valuation of options at hand by integrating various types of information, such as the amount of money one can win and the corresponding probability of winning it, to form a perception of expected value and risk^9^.

Under the CPT framework, our finding that downregulation of the left DLPFC affects probability weighting is consistent with the results of previous studies showing that DLPFC activity correlates with probability distortions without making a choice^15^, while in case of making an active choice DLPFC may play a key role in accumulating reward probability information^65^. The left middle frontal gyrus demonstrated a higher BOLD response when participants chose a low-probability rather than high-probability outcome^66^, suggesting that information on reward probability is more actively processed by the left rather than right DLPFC.

Other possible ways of the DLPFC involvement in risky choice may include its link to the dopaminergic reward system. Recent research suggests that dopamine is a key neurotransmitter implicated in risk-taking behavior^67,68^. Specifically, dopaminergic neurons may encode expected reward as well as reward prediction error in human and animal subjects^69–71^, e.g., computations that are crucial for deciding whether or not to take a risk. An increase of dopamine is associated with an increase of the propensity to risk^72^. Thus, we speculate that the change in probability weighting following downregulation of the DLPFC with cTBS may result from the disruption of the neural circuit between the DLPFC and the basal ganglia. Prefrontal cortex may play a role in regulating the firing rate of the dopaminergic neurons in the ventral tegmental area (VTA)^73^, and its interaction with VTA may be related to information coding in the prefrontal cortices^74^. It has been shown that in a gamble evaluation context, the activity of the NAcc correlated with the degree of probability distortion^75^. Several studies in human subjects demonstrated that high-frequency rTMS over the DLPFC may lead to an increase in dopamine release in the striatum^76,77^. Stimulation of frontal brain regions in rats with 20 Hz rTMS leads to an increase in extracellular dopamine in the striatum and the nucleus accumbens^78^. Similar results were obtained in a study on human subjects where the DLPFC was stimulated with 10 Hz rTMS^79^. The cTBS protocol applied over the DLPFC affected dopamine release in the striatum during the Montreal Card Sorting Task^80^. An fMRI study^35^ demonstrated an enhancement in reward prediction error coding in the ventral striatum and increased reward sensitivity following cTBS over the left DLPFC. Therefore, the effects of DLPFC stimulation observed in the present study might be due to changes in the dopaminergic reward system.

An alternative view on risky decision making implies the use of various heuristics under which the available information about the options is differently attended to depending on the specific heuristic rule used by a participant. These heuristics might imply using information about the options only partially, paying attention only to outcomes or only to probabilities. For example, if a participant relies on a minimax rule (choosing an option with maximum worst outcome) then probabilities are not attended to at all. As has been recently shown, relying on heuristic rules may be reflected in the parameters of the CPT model^81^. Non-linear probability weighting may then result from attentional biases in the decision-making process^7^. If this is indeed the case, then the observed change in probability weighting following the disruption of the DLPFC with cTBS might have resulted from the change in attentional rather than valuation processes. In favor of the use of heuristics in our study speaks the fact that in our sample the estimated baseline probability weighting parameter is greater than 1. This value corresponds to an S-shaped probability weighting (as opposed to an inverse S-shape found in many other studies), which implies underweighting of small probabilities and overweighting of large probabilities. One possible reason for this non-typical shape of the weighting curve may be the use of an heuristic rule. In particular, in the debriefing stage, some participants indicated that when the probability of one of the outcomes was large enough, they disregarded the probability of an alternative outcome, and chose a lottery bringing the highest amount of money with that large probability. As a result, when probability of the best outcome is not equal to 1 but still large, participants might have behaved as if it was equal to 1. The same logic goes for probabilities close to zero. The use of this heuristic might have shown up as an S-shaped probability weighting curve. Importantly, the S-shaped probability weighting was observed among individual subjects in several previous studies^15,75,82^. Further exploration would be needed to identify the cognitive mechanisms behind probability weighting.

At the same time, one consideration speaking against the hypothesis that TMS over left DLPFC affected attentional processes is that the analysis of the response times did not reveal any differences after the stimulation of either left or right DLPFC relative to sham (see Supplemental Materials). We estimated a linear mixed model of a reaction time in the gain domain, which included dummy variables for the right or left stimulation condition, trial number within a session and order of the session (to account for learning effects), absolute difference in the probabilities of outcomes, a dummy variable of whether a risky option was chosen in a given trial, and the interaction terms of the risky choice dummy with probability differences and with stimulation condition. It also included random effects on the subjects’ level. We found that the reaction time was lower for later trials and sessions compared to earlier, which indicates the learning effect and participants getting more familiar with the task. The reaction time was also lower for trials with higher difference in probabilities, since when the probability of one of the outcomes tends to 1 it becomes easier to make a decision. Finally, it took significantly longer time for participants to choose a riskier lottery compared to safer. However, we do not find any direct effect of stimulation condition on the reaction times compared to sham (p = 0.72 and p = 0.91 for right and left DLPFC stimulation respectively). We also do not observe any significant differences in reaction times for riskier choices compared to safer due to the stimulation (p = 0.52 and p = 0.26 for right and left DLPFC stimulation respectively). This observation suggests that in the gain domain the TMS stimulation did not lead to participants making quicker decisions. Under the hypothesis that reaction times indeed reflect attentional processes, this suggests that the changes in probability weighting parameter were not due to a shift in attention. However, disentangling various explanations of risk preferences was not the goal of the present study and further exploration is needed to answer this question more deeply.

Another possibility is that the cTBS of the DLPFC temporarily knocks out its ability to perform executive functions, which may or may not directly relate to decision making in our experiment. In particular, one explanation of our findings could be that the inhibition of the DLPFC leads to the disturbance in working memory which could interfere with a decision-making task. In our study, this explanation is partially ruled out by the fact that in the experimental task all the necessary information in each trial was available to the participants on the screen at the moment of choice. Previous studies have shown that still working memory may be involved in this case, for example, the working memory capacity may be linked to the rationality of probabilistic judgements^83^. However, if TMS effects on working memory would be the only factor explaining the changes in preference parameters, we would expect to see similar effects in gains as well as in losses. Lack of evidence for this suggests that working memory may not be the only factor involved. Further studies, possibly using online TMS techniques, may help clarify the link between the role of the DLPFC in working memory and risky choice.

Additionally, the TMS procedure might have affected the ability for response monitoring and inhibition of choosing an unpreferred option. However, making more of such “errors” would result in less consistent choices in a lottery task. Partially this effect is captured by the noise parameter (*consistency*) in the structural model, for which we did not observe any significant differences after the stimulation.

In the present study we applied conventional criteria for determining statistical significance. In the hierarchical Bayesian estimation of model parameters, we considered the change in a parameter to be credibly different from zero if zero was not included into the 95% CI of the posterior samples. Additionally, we calculated the Bayes Factor for each parameter of interest, considering BF < 3 as indicating anecdotal evidence for the presence of an effect. However, we also observed that the right DLPFC stimulation had an effect on risk aversion parameter which was different from zero at 89% credibility level, a threshold frequently suggested in the Bayesian analysis^50,51^. This change would correspond to an increase in the marginal value of money following the right DLPFC stimulation which would lead to a more risky behavior. This finding would be in line with previous studies where the right DLPFC was implicated in risky choice^9^. Further studies employing lottery choice questions with greater variability in monetary reward values might be able to clarify the causal involvement of the right DLPFC in risk taking behavior.

The fact that no behavioral effects of the DLPFC TMS were observed in our experiment in the loss domain is consistent with the fMRI meta-analysis which showed that DLPFC is more likely to be activated when only gains are possible^9^. This is in line with the hypothesis that separate neural networks are involved in the valuation of gains and losses. For example, the anticipation of monetary gains and losses evokes positive or negative affect, which is correlated with the activation of distinct brain regions^84^. Activity in distinct brain regions may also represent gain- and loss-related expected value of a lottery^85^. Behavioral adjustment in the conditions of reward and punishment may be linked to the activity in different neural circuits^86^.

In light of the above-mentioned roles that the DLPFC might play in risky choice we cannot unequivocally interpret a change in probability weighting parameter as a change in perception of outcome probabilities. The only change we observe is that probabilities affect the decision to take a risk in a more distorted way after the left DLPFC TMS compared to sham.

In this study, we used parametric methods to elicit subjects’ risk preferences. Parametric methods inevitably impose restrictions on the functional form of utility and probability weighting, which may not accurately represent the actual risk preferences of the participants. We used only one-parameter probability weighting functions. In economic literature two-parametric functions are often used as well, which may allow for a distinction between separate components of probability weighting. For example, in the 2-parametric Prelec function, one parameter may be interpreted as the degree of probability distortion (curvature) while the other is interpreted as elevation (overall optimism or pessimism)^87^. However, in order to reliably estimate two-parametric functions a greater number of trials in the experimental task would have been needed, which might lead to fatigue effects. Future studies may overcome this limitation by using non-parametric methods of eliciting risk preferences. Second, in this study we employed a within-subjects design. This design allowed for perfect matching between participants in each stimulation condition. However, it also meant that each subject performed the experimental task three times. As a result, participants may become bored by the end of the experiment and paid less attention to the task at hand in the third session as opposed to the first one. This limitation was partially overridden by randomizing and counterbalancing the stimulation conditions.

It should be mentioned that in the present study we used sham stimulation only on the right DLPFC as a control for placebo effects. In an ideal design, sham on the left DLPFC could also be used to make sure that placebo effects are not lateralized. However, this would imply repeating the same task in 4 rather than 3 experimental sessions causing greater learning effects, boredom and higher attrition rates. To avoid these unwanted consequences we restricted the number of sessions to 3 and applied placebo stimulation only on the right DLPFC. The choice of the right hemisphere was made based on the previous fMRI studies showing that the right DLPFC was more frequently active in decisions under risk. Therefore, the main TMS effect was expected to be seen after the right DLPFC stimulation. Interestingly, a significant change in risky behavior was instead observed after the left DLPFC stimulation. Because the real stimulation was applied both on the left and on the right DLPFC but the right DLPFC did not produce significant changes on behavioral or parametric level, this suggests that the changes in behavior due to unpleasantness of the TMS procedure are partially controlled for. However, future studies targeting specifically exploration of the left DLPFC involvement in risky choice would be needed to properly control for these effects.

To sum-up, our study for the first time shows causal evidence that DLPFC may be involved in probability weighting during risky choice. These findings are consistent with the notion that the DLPFC participates in the neural circuit involved in making decisions under risk. Particularly, we provide weak evidence that downregulation of the DLPFC may change the way outcome probabilities affect the final decision to take a risk.

## Supplementary Information

Supplementary Information.

## Data Availability

Complete dataset used in this study as well the custom code in R and Stan used to analyze the data are freely available in the repository: https://github.com/neuroexperiments/risky_choice_DLPFC_TMS.
