# Supplementary material for: Dorsolateral prefrontal cortex plays causal role in probability weighting during risky choice
Source: Sci Rep. 2022 Sep 27;12:16115. doi: 10.1038/s41598-022-18529-6 (PMC9515118; doi:10.1038/s41598-022-18529-6)
Supplement: Supplementary file 1 — Supplementary Information. [file 41598_2022_18529_MOESM1_ESM.pdf]

Dorsolateral prefrontal cortex plays causal role in probability weighting during risky choice

Authors: Ksenia Panidi*, Alicia Nunez Vorobiova, Matteo Feurra, Vasily Klucharev

Supplemental Material

Complete list of lotteries in experimental task

	Option A		Option B					
Number of question	Outcome 1	Outcome 2	Outcome 3	Outcome 4	Probability of best outcome	Probability of worst outcome	Expected value of Option A	Expected value of Option B
**MPL 1**								
1	125	100	220	55	0	1	100	55
2	125	100	220	55	0.01	0.99	100.25	56.65
3	125	100	220	55	0.05	0.95	101.25	63.25
4	125	100	220	55	0.10	0.90	102.5	71.5
5	125	100	220	55	0.15	0.85	103.75	79.75
6	125	100	220	55	0.20	0.80	105	88
7	125	100	220	55	0.30	0.70	107.5	104.5
8	125	100	220	55	0.40	0.60	110	121
9	125	100	220	55	0.50	0.50	112.5	137.5
10	125	100	220	55	0.60	0.40	115	154
11	125	100	220	55	0.70	0.30	117.5	170.5
12	125	100	220	55	0.80	0.20	120	187
13	125	100	220	55	0.85	0.15	121.25	195.25
14	125	100	220	55	0.90	0.10	122.5	203.5
15	125	100	220	55	0.95	0.05	123.75	211.75
16	125	100	220	55	1	0	125	220
**MPL 2**								
1	260	180	350	50	0	1	180	50
2	260	180	350	50	0.01	0.99	180.8	53
3	260	180	350	50	0.05	0.95	184	65
4	260	180	350	50	0.10	0.90	188	80
5	260	180	350	50	0.15	0.85	192	95
6	260	180	350	50	0.20	0.80	196	110
7	260	180	350	50	0.30	0.70	204	140
8	260	180	350	50	0.40	0.60	212	170
9	260	180	350	50	0.50	0.50	220	200
10	260	180	350	50	0.60	0.40	228	230
11	260	180	350	50	0.70	0.30	236	260
12	260	180	350	50	0.80	0.20	244	290
13	260	180	350	50	0.85	0.15	248	305
14	260	180	350	50	0.90	0.10	252	320
15	260	180	350	50	0.95	0.05	256	335
16	260	180	350	50	1	0	260	350
**MPL 3**								
1	230	110	335	10	0	1	110	10
2	230	110	335	10	0.01	0.99	111.2	13.25
3	230	110	335	10	0.05	0.95	116	26.25
4	230	110	335	10	0.10	0.90	122	42.5
5	230	110	335	10	0.15	0.85	128	58.75
6	230	110	335	10	0.20	0.80	134	75
7	230	110	335	10	0.30	0.70	146	107.5
8	230	110	335	10	0.40	0.60	158	140
9	230	110	335	10	0.50	0.50	170	172.5
10	230	110	335	10	0.60	0.40	182	205

11	230	110	335	10	0.70	0.30	194	237.5
12	230	110	335	10	0.80	0.20	206	270
13	230	110	335	10	0.85	0.15	212	286.25
14	230	110	335	10	0.90	0.10	218	302.5
15	230	110	335	10	0.95	0.05	224	318.75
16	230	110	335	10	1	0	230	335

Table S-1. Multiple Price Lists used in the experimental task (the same absolute amounts were used for lotteries in the loss domain):

Parameter recovery

Parameter recovery was performed for the model which showed better fit on the real data, i.e. the model with Kahneman-Tversky probability weighting function in the gain domain. For parameter recovery, we simulated datasets across various parameter values and its combinations. Following the recommendations in (Wilson and Collins 2019), the values were selected in the same range as the values obtained from the real data analysis. The estimated model contains 9 group-level parameters (3 baseline parameters, 3 parameters indicating the effects of the right DLPFC stimulation and 3 parameters for the effects of the left DLPFC stimulation) for which the recoverability was tested. If each parameter is assigned 3 possible values, a fully-crossed design would imply 19683 possible permutations and corresponding simulated datasets. To reduce the number of simulated datasets we fixed the values of the baseline parameters and varied the values of the main parameters of interest, i.e. the effects of stimulation on risk aversion, probability weighting and consistency. The following group-level parameter values were chosen for the recovery procedure: $\mu_{r^0} = 1, \mu_{\gamma^0} = 2.5, \mu_{\tau^0} = 7, \mu_{\Delta r^{right}} = (0.1, 0.2, 0.3)$, $\mu_{\Delta\gamma^{right}} = (0.3, 0.5, 0.7)$, $\mu_{\Delta\tau^{right}} = (-0.5, 0, 0.5)$, $\mu_{\Delta r^{left}} = (0.2, 0.4, 0.6)$, $\mu_{\Delta\gamma^{left}} = (0.4, 0.6, 0.8)$, $\mu_{\Delta\tau^{left}} = (-1, 0, 1)$. For each of the possible 729 permutations of these parameter values we generated a combination of 28 individual parameter values assuming that individual parameters are distributed normally with the mean equal to the corresponding group-level parameter value and the standard deviation equal to the standard deviation obtained from the experimental data individual estimates (numbers are rounded): $\sigma_{r^0} = 0.58$, $\sigma_{\gamma^0} = 1.46$, $\sigma_{\tau^0} = 2.11$, $\sigma_{\Delta r^{right}} = 0.03$, $\sigma_{\Delta\gamma^{right}} = 0.42$, $\sigma_{\Delta\tau^{right}} = 0.59, \sigma_{\Delta r^{left}} = 0.12$, $\sigma_{\Delta\gamma^{left}} = 0.24, \mu_{\Delta\tau^{left}} = 1.79$.

To avoid sampling unrealistic individual parameter values the normal distributions for each parameter were truncated at 0 on the left and the distribution mean multiplied by 2 on the right. After generating the 729 sets of 28 individual parameters, for each synthetic subject we simulated the choices in the experimental task structured exactly in the same way as in the real experiment creating 48*3 binary choices for each subject. Each of the

729 artificially created datasets was then submitted to the same estimation pipeline as the one used to analyze real experimental data.

We then calculated the difference between the obtained group-level parameter estimates and the generating values for each simulated dataset. Figure S-1 shows the estimation bias for each model parameter.

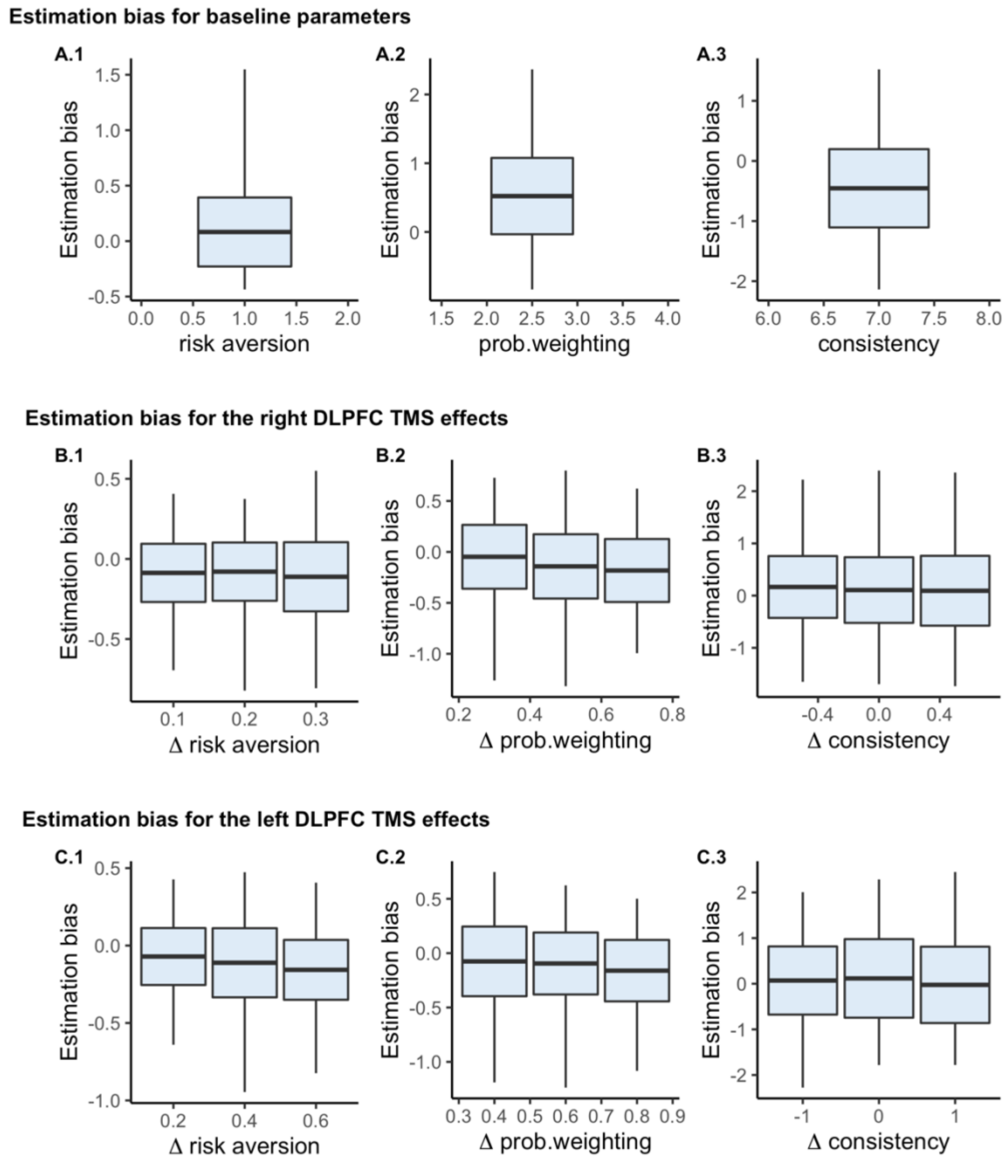

Figure S-1. Estimation bias as the difference between recovered and generating parameter values. Each boxplot represents mean±SD, as well as min and max for each parameter estimation bias.

Although recovery of the baseline parameters μ_{γ^0} and μ_{τ^0} is less precise, parameter μ_{r^0} and, more importantly, parameters indicating the effects of TMS can be reliably estimated. Additionally, we calculated the rate with which the true parameter value was contained in the 95% credible interval. The obtained recovery rates were as follows: μ_{r^0} 0.95,

μ_{γ^0} 0.82, μ_{τ^0} 0.88, $\mu_{\Delta r^{right}}$ 0.90, $\mu_{\Delta\gamma^{right}}$ 0.91, $\mu_{\Delta\tau^{right}}$ 0.94, $\mu_{\Delta r^{left}}$ 0.91, $\mu_{\Delta\gamma^{left}}$ 0.95, $\mu_{\Delta\tau^{left}}$ 0.95. For all parameters measuring the TMS effects the recovery rate was higher than 90%.

Robustness check: analysis including all 30 subjects

As a robustness check we performed the same behavioral analysis and modelling of risk preferences without excluding 2 subjects with large interhemispheric difference in motor threshold. One of these subjects still could not be included in the regression analysis due to missing data on self-reported level of discomfort. However, including one other subject did not affect the results. Regression results are presented in Table S-2.

Dependent variable:	Probability of choosing a riskier lottery		Probability of choosing a higher EV lottery	
	gains	losses	gains	losses
TMS (right DLPFC)	-0.047	0.018	0.021	0.029
	(0.241)	(0.218)	(0.238)	(0.245)
TMS (left DLPFC)	-0.057	-0.0005	-0.521*	-0.346
	(0.242)	(0.218)	(0.225)	(0.229)
Discomfort	-0.139	0.142	-0.173*	-0.127
	(0.093)	(0.084)	(0.086)	(0.084)
Trial	-0.001	0.001	-0.0005	-0.001
	(0.002)	(0.002)	(0.002)	(0.002)
Difference in std.dev.	0.022***	0.006***	-0.037***	-0.028***
	(0.002)	(0.001)	(0.002)	(0.002)
Difference in means	0.015***	0.010***	0.0003	0.0002
	(0.002)	(0.001)	(0.002)	(0.002)
TMS (right DLPFC) x Discomfort	0.092	-0.075	0.089	0.027
	(0.103)	(0.093)	(0.100)	(0.102)
TMS (left DLPFC) x Discomfort	0.107	-0.093	0.282**	0.130
	(0.097)	(0.088)	(0.092)	(0.091)
Difference in std.dev. x Difference in means	0.0005***	0.0004***	-0.0002***	-0.0001**
	(0.00003)	(0.00002)	(0.00003)	(0.00003)
Observations	4,176	4,176	4,176	4,176
Log Likelihood	-1,341.340	-1,625.437	-1,465.005	-1,401.988
Akaike Inf. Crit.	2,704.680	3,272.874	2,952.009	2,825.975

Table S-2. Effects of the right/left DLPFC TMS on the behaviour in a binary lottery choice task on a trial-by-trial level relative to sham. All regressions are mixed-effect generalized linear models with a logit link function and subject-level random effects. Columns 1 and 2 use probability of choosing a riskier (higher SD) lottery as a dependent variable. Columns 3 and 4 use probability of choosing a lottery with higher expected reward as a dependent variable. *** p < 0.001; ** p < 0.01; * p < 0.05. Standard errors in parentheses.

We also estimated the model of risk preferences on all 30 subjects in the gain domain (**Table S-3**).

Parameter	Mean	89% CI	95% CI	BF
Effects of the right DLPFC TMS				
Δ risk aversion ($\mu_{\Delta r^{right}}$)	0.211	[0.002 ; 0.41]	[-0.002 ;0.47]	0.34
Δ prob. weighting ($\mu_{\Delta \gamma^{right}}$)	0.322	[-0.07 ; 0.84]	[-0.16 ; 0.98]	0.35
Δ consistency ($\mu_{\Delta \tau^{right}}$)	0.417	[-0.71 ; 1.69]	[-0.96 ; 2.10]	0.19
Effects of the left DLPFC TMS				
Δ risk aversion ($\mu_{\Delta r^{left}}$)	0.127	[-0.08; 0.37]	[-0.12 ; 0.45]	0.15
Δ prob. weighting ($\mu_{\Delta \gamma^{left}}$)	0.513	[0.06 ; 1.02]	[0.003; 1.14]	0.82
Δ consistency ($\mu_{\Delta \tau^{left}}$)	0.699	[-0.87 ; 2.44]	[-1.22; 2.85]	0.99

Table S-3. Summary the DLPFC TMS effects on risk preference parameters in the gain domain: mean, 89% and 95% CIs, and the Bayes Factor. The Bayes Factor indicates evidence in favor of $H_1: \delta \neq 0$ against $H_0: \delta = 0$.

The results do not substantially differ from those based on 28 participants, according to the 95% CI criterion. At the same time, Bayes factor for testing the hypothesis that the parameter of interest differs from zero, does not indicate significant change in probability weighting after the left DLPFC stimulation. However, given that the effect of the left DLPFC stimulation is evaluated in comparison to the baseline determined by the sham stimulation on the right DLPFC, this result suggests that the inclusion of two participants with large left-right excitability asymmetry indeed makes evidence less reliable.

Modelling risk preference parameters in the loss domain

At the baseline, participants were risk averse in losses at the group level with mean risk aversion estimated at μ_{r^0}= 1.81, 95% CI = [1.42, 2.26]. On the individual level, 11

participants demonstrated risk seeking, while 17 showed risk aversion. Significant distortion of probability was also observed with μ_{γ^0}= 1.38, 95% CI = [1.08, 1.73]. Participants were consistent in their answers as μ_{τ^0}= 8.70, 95% CI = [6.63, 11.2].

Table S-4 presents the means, 89% and 95% CIs, probability of direction, and the Bayes Factor values to characterize changes in risk aversion, probability weighting and consistency in the loss domain. Figure S-2 shows the posterior distributions for the estimated parameters.

Parameter	Mean	89% CI	95% CI	BF
Effects of the right DLPFC				
Δ risk aversion ($\mu_{\Lambda_r^{right}}$)	0.01	[-0.17 ; 0.19]	[-0.21 ; 0.23]	0.06
Δ prob. weighting ($\mu_{\Lambda_\gamma^{right}}$)	-0.007	[-0.16 ; 0.13]	[-0.2 ; 0.17]	0.07
Δ consistency ($\mu_{\Lambda_\tau^{right}}$)	1.473	[-0.43 ; 3.61]	[-1.38 ; 4.11]	0.86
Effects of the left DLPFC				
Δ risk aversion ($\mu_{\Lambda_r^{left}}$)	0.158	[-0.01 ; 0.34]	[-0.04 ; 0.39]	0.20
Δ prob. weighting ($\mu_{\Lambda_\gamma^{left}}$)	0.104	[-0.06 ; 0.28]	[-0.09 ; 0.33]	0.14
Δ consistency ($\mu_{\Delta_\tau^{left}}$)	1.202	[-0.98 ; 3.73]	[-1.45 ; 4.2]	0.44

Table S-4. Summary the DLPFC TMS effects on risk preference parameters in the loss domain: mean, 89% and 95% CI, and the Bayes Factor. The Bayes Factor indicates evidence in favor of $H_1: \delta \neq 0$ against $H_0: \delta = 0$.

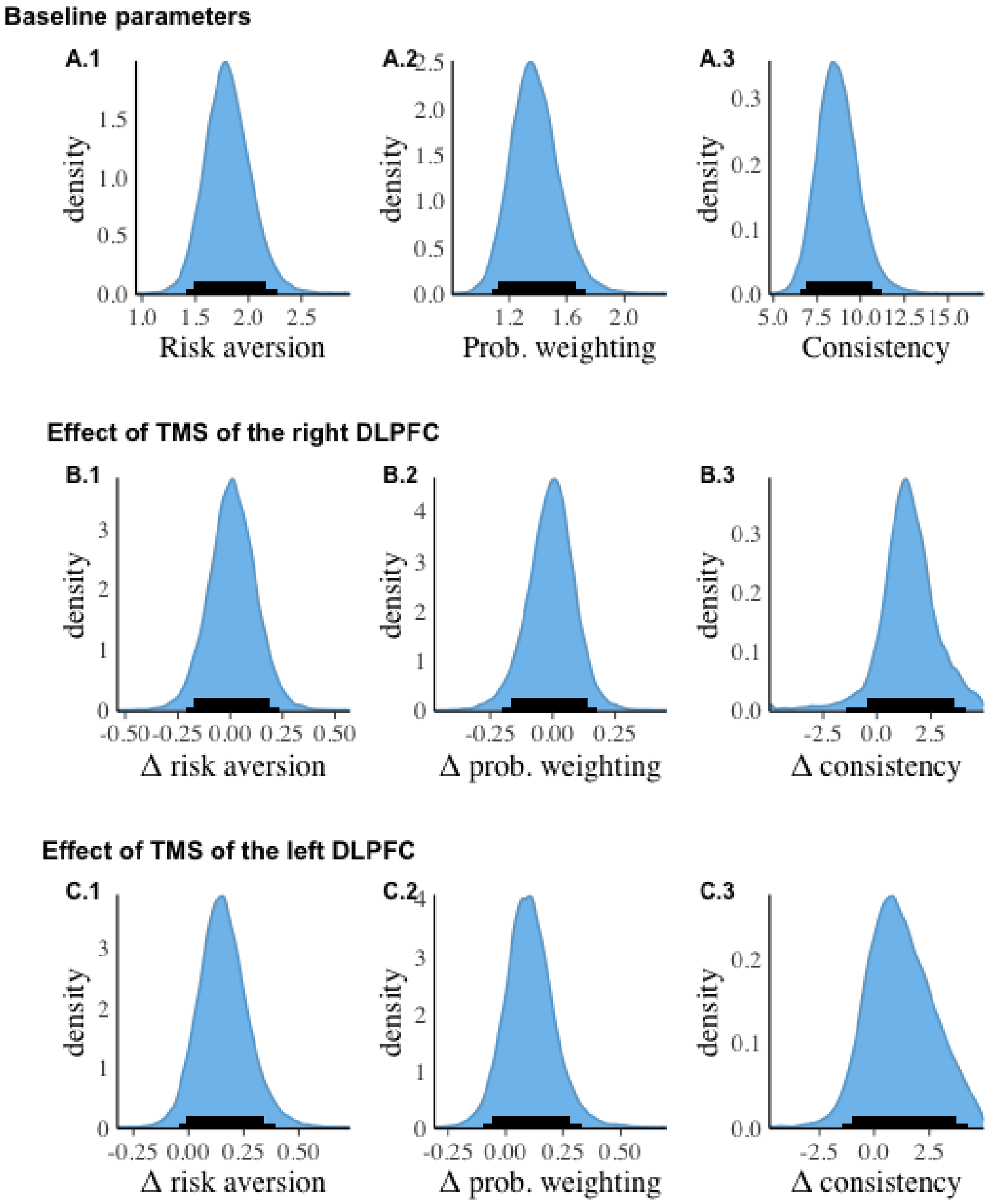

Figure S-2. Baseline risk preference parameters and their changes after the DLPFC TMS in the loss domain. Baseline risk preference parameters: A.1 risk aversion (μ_{r^0}), A.2 probability weighting (μ_{γ^0}), A.3 consistency (μ_{τ^0}). Shift in risk preference parameters after right DLPFC TMS: B.1 change in risk aversion ($\mu_{\Delta r^{right}}$); B.2 change in probability weighting ($\mu_{\Delta\gamma^{right}}$); B.3 change in consistency ($\mu_{\Delta\tau^{right}}$). Shift in risk preference parameters after left DLPFC TMS: C.1 change in risk aversion ($\mu_{\Delta r^{left}}$); C.2 change in probability weighting ($\mu_{\Delta\gamma^{left}}$); C.3 change in consistency ($\mu_{\Delta\tau^{left}}$). The thin and thick black lines on the horizontal axis indicate the 95% and 89% CIs respectively.

In the loss domain, downregulation of the left or right DLPFC did not produce any change in risk preference parameters.

Analysis of TMS effects on reaction times

	Dependent variable: reaction time (ms)
Session order	**-1319.54 *****
	(82.54)
Trial	**-18.51 *****
	(2.42)
Dummy (riskier lottery chosen)	**1446.54 *****
	(400.26)
TMS (right DLPFC)	74.35
	(207.96)
TMS (left DLPFC)	24.17
	(207.72)
Abs. difference in probabilities	**-2249.30 *****
	(270.19)
Gender	1214.45
	(834.54)
Dummy (risky lottery chosen) : TMS (right DLPFC)	-218.58
	(341.92)
Dummy (risky lottery chosen) : TMS (left DLPFC)	-388.91
	(342.32)
Dummy (risky lottery chosen) : Abs. difference in probabilities	**-1530.21 ****
	(479.62)
N	4032
N (subject_ID)	28
AIC	78836.19

Table S-5. Linear mixed model for reaction time as a dependent variable with subject level random effects. *** $p < 0.001$; ** $p < 0.01$; * $p < 0.05$. Standard errors in parentheses.

References:

Wilson, Robert C, and Anne GE Collins. 2019. "Ten Simple Rules for the Computational Modeling of Behavioral Data." *ELife* 8 (November). https://doi.org/10.7554/eLife.49547.
